# Emergency Cricothyrotomy in a Harsh Environment: A Case Report of Complete Airway Obstruction Following a Lightning Strike

**DOI:** 10.1213/XAA.0000000000001688

**Published:** 2023-06-19

**Authors:** Barbara Pizzi, Emiliano Petrucci, Franco Marinangeli

**Affiliations:** From the * Department of Anesthesia and Intensive Care Unit, SS Filippo and Nicola Academic Hospital of Avezzano, L’Aquila, Italy; † Department of Anesthesia and Intensive Care Unit, San Salvatore Academic Hospital of L’Aquila, L’Aquila, Italy; ‡ Department of Life, Health and Environmental Sciences, University of L’Aquila, L’Aquila, Italy.

## Abstract

A lightning strike is an extreme event with the highest mortality rate among electrical injuries. Death from a lightning strike is caused by either cardiac arrest or respiratory arrest. It is rare for upper airway damage to occur, but in these cases, airway control is recommended. If transoral intubation is unsuccessful, an emergency cricothyrotomy should be considered. Our case report describes an emergency cricothyroidotomy performed in a harsh environment on a mountain 2300 m above sea level on a patient with extensive burns of his supraglottic structures, after being directly hit by a lightning strike.

A lightning strike is an extreme event with the highest mortality rate among electrical injuries.^1^

The ventilatory center in the brain stem and the heart is particularly vulnerable to electric injuries. Consequently, death is caused by either a cardiac arrest or a respiratory arrest. Although in many cases the intrinsic cardiac automaticity may spontaneously restore organized cardiac activity and a perfusing rhythm, the concomitant ventilatory arrest may continue after the return of spontaneous circulation (ROSC).^2^ Thus, control of the airway to ensure adequate oxygenation and ventilation is recommended to avoid a secondary hypoxic cardiac arrest.^3^ The standard of care remains transoral intubation,^4,5^ but in case of failure, an emergency cricothyrotomy is indicated if the situation can rapidly prove fatal.

This case report was approved by our institutional review board, and written informed patient consent was obtained. This article adheres to the applicable CAse REports (CARE) guidelines.

## REPORT OF THE CASE

On August 27, 2022, 3 hikers were hit by a stroke of lightning at 2300 m above sea level, on the Gran Sasso Mountain chain (L’Aquila, Italy).^6^ Two of them were indirectly hit and were not critically injured. According to bystanders, a 26-year-old man was directly hit on the head. He fell down, and he was unconscious but had carotid pulses. The other two hikers also testified seeing smoke coming out of the third hiker’s mouth.

The helicopter emergency medical service of L’Aquila was immediately activated. Five minutes after the activation, the rescue team reached the target during a thunderstorm with hail and lightning. The rescue team consisted of an anesthesiologist, a nurse, 2 pilots, and a hoister man.

**Figure 1.**
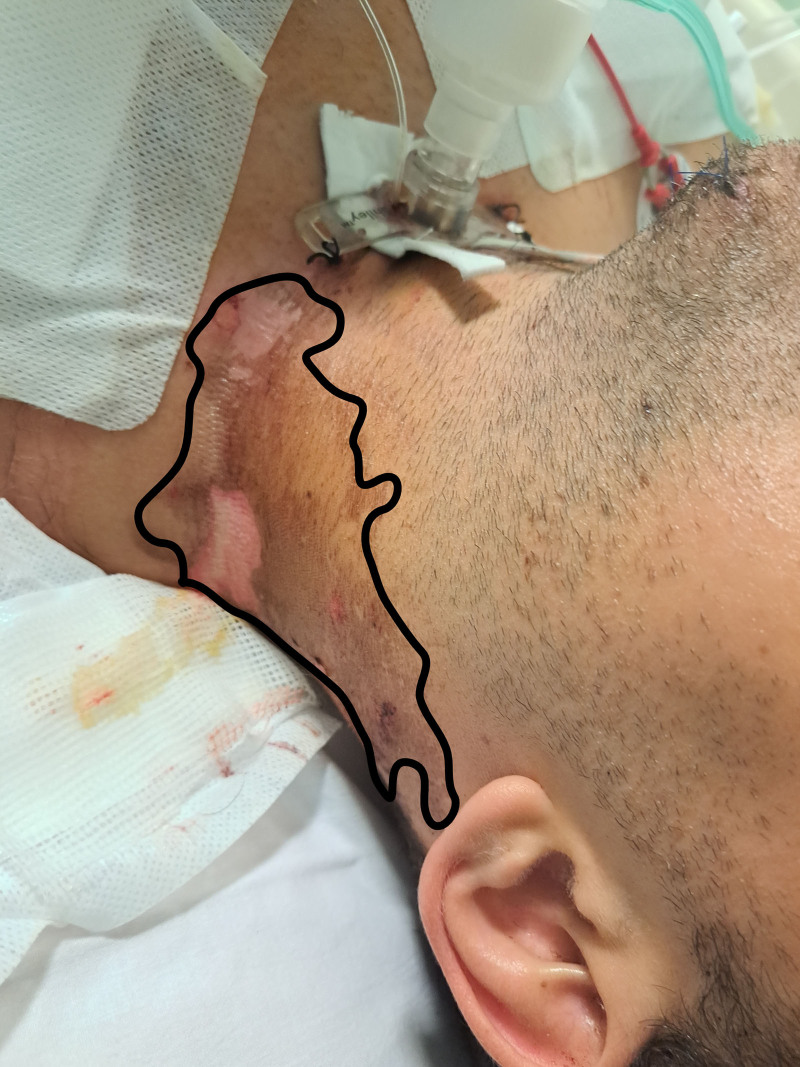
Burn injuries on the left side of the neck (black line).

The patient had palpable carotid pulses, and his heart rate was 135 beats·min^–1^. Radial, brachial, and femoral pulses were not palpable. Peripheral capillary oxygen saturation (Spo_2_) and arterial blood pressure could not be assessed. The electrocardiogram was not performed, due to the risk of the electrostatic discharge. The Glasgow Coma Scale was 5 (eye-opening response: 1; verbal response: 1; and motor response: 3). The patient’s respiratory rate was 6 breaths min^–1^, but without gasping. Burn injuries were recognizable on the left side of the neck (Figure 1), chest wall, legs, and feet. Blast injuries were found on his lips and chin, while Lichtenberg figures were present on the right side of the neck and back, and along the spinal processes.

Then, the patient’s respiratory rate was further dropping, and his ventilatory efforts were more shallow. Ventilation and oxygenation using a self-inflating bag and a supraglottic airway were not possible. Thoracic and limb muscle spasms and trismus were observed. Subsequently, 90 mg of propofol was intravenously administered to evaluate the airway by laryngoscopy and to facilitate orotracheal intubation. Neuromuscular blocking agents were not used.^7^

The mouth and hypopharynx were suctioned. Direct laryngoscopy using a Macintosh blade revealed edema and burns of the epiglottis, vestibular folds, and arytenoids. In addition, orotracheal secretions and blood clots completely occluded his mouth, oral cavity, and tracheal lumen.

Three unsuccessful orotracheal intubation attempts were performed, including the use of a stylet-guided tracheal tube insertion and a video laryngoscope.

An emergency cricothyroidotomy (Melker Emergency Cricothyrotomy Catheter Set [Seldinger], COOK MEDICAL LLC) was performed in the field by puncturing the cricothyroid membrane with an 18 gauge needle attached to a 10 mL syringe half filled with saline. The needle was advanced through the skin of the neck, while applying continuous negative pressure on the syringe. The air bubbles visualized in the syringe confirmed the needle placement within the trachea. Once the needle was within the trachea, a guidewire was inserted through the needle (Seldinger technique). A dilator was inserted over the guidewire to facilitate the insertion of the endotracheal tube (inside diameter: 5 mm) included in the kit. The self-inflating bag confirmed placement of the endotracheal tube with end-tidal capnography.

No procedural complications were noted.

Suddenly, the patient went into cardiac arrest, and prompt advanced cardiovascular life support was started. External chest-wall compression was performed, and 1 mg of epinephrine was intravenously administered. After 5 minutes, ROSC with palpable carotid pulses was obtained, and the patient was transported via helicopter to the hospital.

**Figure 2.**
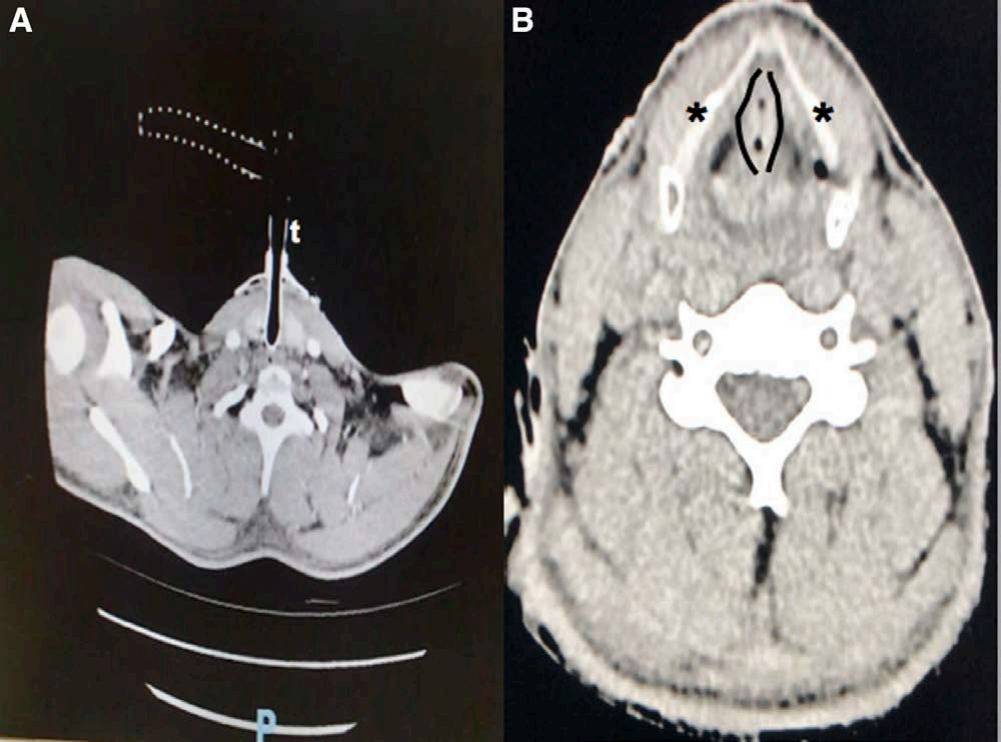
Computed tomography scan. A, Correct placement of the tube (t) within the lumen of the trachea. B, Hypopharynx and oropharynx full-thickness obstruction with a complete narrowing of the supraglottic space: edema of vocal cords (black lines) and soft tissue behind the thyroid cartilage (black stars).

In the hospital, the Spo_2_ was 98%, heart rate was 125 beats min^–1^, and arterial blood pressure was 85/40 mm Hg. A computed tomography (CT) scan confirmed the correct placement of the tube within the lumen of the trachea (Figure 2A). Hypopharyngeal and oropharyngeal full-thickness edema with a complete narrowing of the supraglottic space was documented (Figure 2B). The intravenous contrast media used to enhance CT imaging revealed hyperemia of the epiglottis, and edema of the vocal cords and soft tissue behind the thyroid cartilage.

The patient was admitted to the intensive care unit (ICU). An open surgical tracheostomy was performed at level of the third tracheal ring, removing the endotracheal tube placed during the emergency cricothyroidotomy. Sedative medications were administered, and mechanical ventilation was initiated. Prophylactic multiple doses of dexamethasone (16 mg day^–1^ in 2 divided doses) were administered to decrease the risk of postdecannulation laryngeal and tracheal edema.^8^

Four days later, he was weaned from mechanical ventilation with no neurological impairment and no need for cardiovascular drug support. Seven days after weaning, a fiberoptic evaluation documented a patent airway and allowed complete decannulation.

## DISCUSSION

A lightning stroke is a special case of electrical injury. The exposure time is very short with a duration usually not exceeding 200 ms, and the energy level is extremely high (voltages ranging up to 100,000,000 volts).

Direct high-voltage upper airway damages have been described in a small number of case reports.^1^ Acute airway management remains a life-threatening challenge following lightning strikes. Victims of a lightning strike may rapidly develop acute airway obstruction, due to electric burns on the face, mouth, or neck and soft-tissue swelling. Advanced management of the airway including early endotracheal intubation should be performed even if the patient is spontaneously breathing.^2^ In the harsh environment of mountains, it is essential to identify a “cannot ventilate/cannot intubate” situation and perform an emergency cricothyrotomy when other maneuvers have failed to avoid cardiac and respiratory arrest.^5^

According to Critchley’s suggestions, several mechanisms contribute to the airway injuries caused by lightning strikes.^9^ The lightning current passes through the respiratory centers such as the brain stem, pons, and medulla, resulting in central hypoventilation progressing to ventilatory arrest.^9^ The current primarily passes through the blood vessels.^9^ This may lead to high-voltage burns and acute microvascular embolism with necrosis and thrombosis. It is also possible that a single stroke of lightning can quickly heat the air around it to 30,000 °C, explosively and rapidly expanding gases.^10^ The gas expansion creates a “microdetonation” with a shock wave in the upper airways. The resulting barotrauma may lead to rupture of the mucosa, and hyperacute inflammatory responses with reactive hyperemia of the subepithelial capillary network of the epiglottis.^10^

We speculate that the oropharyngeal and hypopharyngeal mucosa directly exposed to the current of the lightning strike rapidly developed a hyperinflammatory response with mucosal architecture disruption, submucosal tissue swelling, and necrosis, as was observed during the orotracheal intubation attempts.

In our case, we assume that the full-thickness burns and edema of the supraglottic structures seen on the CT images were directly caused by the high-voltage energy from the lightning strike (Figure 2A, B). Thus, orotracheal intubation was not feasible, and the cardiac arrest was due to the secondary transient hypoxemia. In fact, before the endotracheal tube was inserted, the Spo_2_ was not assessable, and peripheral pulses were not palpable. After the emergency cricothyroidotomy was performed, the patient’s oxygenation and ventilation were reestablished, and oxygen delivery and arterial oxygen content were restored.

Propofol was postulated to increase the brain’s tolerance to hypoxia and cerebral ischemia.^11^ This might play an important role in neuroprotection when ventilation and oxygenation are limited. The avoidance of neuromuscular blocking agents to perform orotracheal intubation is associated with increased difficulty or failed intubation.^12^ However, if transoral intubation is unsuccessful, the pharmacological reversal of muscle relaxants agents does not guarantee a prompt respiratory recovery. This can lead to dangerous periods of hypoxemia.

The helicopter emergency medical team of L’Aquila is equipped with a wire-guided cricothyrotomy kit. There is some controversy over which cricothyrotomy technique is superior. The wire-guided procedure has been shown to be more reliable, leading to fewer complications compared to the cannula-over-needle method.^13^

In conclusion, we believe that the emergency cricothyroidotomy performed on the field allowed the rescue team to safely and quickly evacuate the victim from the harsh environment of the mountains and the bad weather conditions, thereby managing an otherwise inaccessible airway and possibly avoiding brain injury.^2^

## ACKNOWLEDGMENTS

We acknowledge Gioele Marrocco, Giacomo Sollecchia, and all staff of “Luca Tonini” Simulation Center, Department of Life, Health and Environmental Sciences, University of L’Aquila, L’Aquila, Italy.

## DISCLOSURES

**Name**: Barbara Pizzi, MD.

**Contribution**: This author recruited patients, conceived the study, designed the report, drafted the manuscript, and contributed substantially to its revision.

**Name**: Emiliano Petrucci, MD.

**Contribution**: This author drafted the manuscript and contributed substantially to its revision.

**Name**: Franco Marinangeli, MD, PhD.

**Contribution**: This author takes responsibility for the paper as a whole, and contributed substantially to its revision.

**This manuscript was handled by:** BobbieJean Sweitzer, MD, FACP.
